# The basic chemistry of exercise-induced DNA oxidation: oxidative damage, redox signaling, and their interplay

**DOI:** 10.3389/fphys.2015.00182

**Published:** 2015-06-17

**Authors:** James N. Cobley, Nikos V. Margaritelis, James P. Morton, Graeme L. Close, Michalis G. Nikolaidis, John K. Malone

**Affiliations:** ^1^Division of Sport and Exercise Sciences, Abertay UniversityDundee, UK; ^2^Exercise Physiology and Biochemistry Laboratory, School of Physical Education and Sport Sciences at Serres, Aristotle University of ThessalonikiSerres, Greece; ^3^Muscle Metabolism Research Group, Research Institute for Sport and Exercise Science, Liverpool John Moores UniversityLiverpool, UK

**Keywords:** DNA damage, redox signaling, exercise, hydrogen peroxide, hydroxyl radical, mitochondrial DNA damage

## Abstract

Acute exercise increases reactive oxygen and nitrogen species generation. This phenomenon is associated with two major outcomes: (1) redox signaling and (2) macromolecule damage. Mechanistic knowledge of how exercise-induced redox signaling and macromolecule damage are interlinked is limited. This review focuses on the interplay between exercise-induced redox signaling and DNA damage, using hydroxyl radical (^·^OH) and hydrogen peroxide (H_2_O_2_) as exemplars. It is postulated that the biological fate of H_2_O_2_ links the two processes and thus represents a bifurcation point between redox signaling and damage. Indeed, H_2_O_2_ can participate in two electron signaling reactions but its diffusion and chemical properties permit DNA oxidation following reaction with transition metals and ^·^OH generation. It is also considered that the sensing of DNA oxidation by repair proteins constitutes a non-canonical redox signaling mechanism. Further layers of interaction are provided by the redox regulation of DNA repair proteins and their capacity to modulate intracellular H_2_O_2_ levels. Overall, exercise-induced redox signaling and DNA damage may be interlinked to a greater extent than was previously thought but this requires further investigation.

## Introduction

Acute exercise disrupts homeostasis, imposing a transient stress that inducts beneficial cyto-protective responses and adaptations with repeated bouts (Cobley et al., 2012; Egan and Zierath, 2013; Hawley et al., 2014). One key homeostatic perturbation is the exercise-induced increase in reactive oxygen (ROS) and nitrogen (RNS) species generation (Cobley et al., 2014). The exercise-induced increase in ROS/RNS generation is bi-functional causing cellular damage and inducting redox signaling (Powers and Jackson, 2008; Cobley et al., 2015a,b). This bi-functionality explains how acute exercise can cause nuclear and mitochondrial DNA oxidation, a mutagenic and damaging event, but exercise training up-regulates DNA repair providing protection against exercise-induced genomic stress (Radak et al., 2011a; Cobley et al., 2013). This synergy between exercise-induced DNA damage and redox signaling whilst conceptually obvious is mechanistically ill-understood. This is compounded by the rare delineation of the species responsible for each outcome in the exercise literature. The purpose of this review and indeed its principal novel feature is to use two exemplar ROS, hydroxyl radical (^·^OH) and hydrogen peroxide (H_2_O_2_), to illustrate the basic chemistry of exercise-induced DNA damage and redox signaling before considering mechanisms that link the two processes together.

## Exercise-induced DNA damage: The key role of hydroxyl radical

From a chemical perspective, superoxide (O_2_^.−^), and nitric oxide (NO), two parent radicals formed during exercise (Sakellariou et al., 2014), do not directly damage DNA (Dizdaroglu and Jaruga, 2012). Analogously, H_2_O_2_ does not directly damage DNA (Halliwell and Gutteridge, 2007). Instead, DNA damage is mediated by ^·^OH and other species capable of modifying DNA including *inter alia*: peroxynitrite, carbonate radical, and nitrogen trioxide (Cadet et al., 2012). In particular, ^·^OH rapidly reacts with DNA bases and the ribose sugar at diffusion-controlled rates (e.g., guanine: *k* ~ 5–8 × 10^9^ M^−1^ s^−1^, Chatgilialoglu et al., 2011). The chemistry of ^·^OH mediated DNA damage is complex but the salient points are: (1) ^·^OH reacts with DNA indiscriminately via addition (*k* ~ 4–9 × 10^9^ M^−1^ s^−1^) or hydrogen abstraction reactions (*k* ~ 2 × 10^9^ M^−1^ s^−1^; Von Sonntag, 2006) and (2) the resultant radical products can then react with other radicals (e.g., O_2_^.−^ and NO) or O_2_ to generate a modified DNA adduct (Dizdaroglu, 2012; Dizdaroglu and Jaruga, 2012). It follows that the chemical identity of the product (s) formed varies according to (1) the type of base modified (2) the nature of the initial reaction (addition or hydrogen abstraction) and (3) levels of secondary reactants (e.g., O_2_^.−^). Accordingly, ^·^OH-DNA reactions yield a multitude of end-products. The oxidation of guanine alone can generate ≥20 end-products, with 8-oxo-7,8-dihydroguanine (8-oxoG) being a frequently assayed end-product owing to its mutagenicity (Radak et al., 2011b).

^·^OH can be generated by the reaction of H_2_O_2_ with transition metals (see below).

*Reaction 1*: H_2_O_2_ + Fe^2+^ → Fe^3+^ + ^−^OH + ^·^OH (*k* ~ 76 M^−1^ s^−1^; Halliwell and Gutteridge, 2007)
*Reaction 2*: H_2_O_2_ + Cu^+^ → Cu^2+^ + ^−^OH + ^·^OH (*k* ~ 4.7 × 10^3^ M^−1^ s^−1^; Halliwell and Gutteridge, 2007)

Acute exercise increases proxies of ^·^OH generation (Close et al., 2005) likely owing to increased H_2_O_2_ generation and disrupted transition metal handling, resulting in increased labile iron and copper (Cobley et al., 2015a). Only vicinal ^·^OH has capacity to damage mitochondrial and nuclear DNA owing to its diffusion-controlled reactivity with cellular biomolecules (Halliwell, 2012). A resultant mechanistic requirement exists for ^·^OH to be generated proximal to DNA and existence of conditions that promote reactions 1 and 2 (e.g., nuclear H_2_O_2_ diffusion) for exercise-induced ^·^OH mediated DNA damage to occur. An additional mechanistic point worthy of consideration is the origin of nuclear and mitochondrial H_2_O_2_ and thus ^·^OH. Mitochondria have a considerably greater capacity to generate H_2_O_2_ internally compared to nuclei, owing to localized O_2_^.−^ generation and subsequent intra-mitochondrial O_2_^.−^ to H_2_O_2_ dismutation capacity (Murphy, 2009, 2012). Exercise-induced nuclear DNA damage likely requires nuclear H_2_O_2_ diffusion from other intracellular sources (e.g., endoplasmic reticulum). The nature of this diffusion is poorly understood yet it is tempting to speculate the existence of retrograde H_2_O_2_ mitochondria-nuclei signaling and damage pathways (see Box 1). It should be noted that present assays, notably assessment of global 8-oxoG levels post-exercise, provide little mechanistic information, owing to the existence of DNA repair processes (Murphy et al., 2011).

Box 1 **A modified mitochondrial bifurcation hypothesis.**

In considering mitochondrial DNA oxidation, the bifurcation hypothesis becomes more complex. Mitochondrial O_2_^.−^ originates from several sources, notably ETC complexes I and III (Murphy, 2009; Finkel, 2011; Goncalves et al., 2015). One fate of mitochondrial O_2_^.−^ is reaction with SOD isoforms to generate H_2_O_2_ (McCord and Fridovich, 1969). The fates of H_2_O_2_ thereafter are numerous and include *inter alia* reaction with: (1) peroxiredoxin 3 and 5 (2) glutathione peroxidase 1 (3) protein thiols (4) labile transition metals or centered proteins (e.g., aconitase) and (5) diffusion out of the mitochondrion (Murphy, 2012). Fates 4 and 5 permit proximal and distal ^·^OH generation and thus damage to mitochondrial and nuclear DNA, respectively. Mitochondria manufacture hem and iron sulfur centered proteins (Collins et al., 2012), which could promote fate 4 and consequent mitochondrial DNA damage. Complexity is added by the O_2_^.−^ source-function-energetic state relationship (Bleier et al., 2015). It is conceivable that some O_2_^.−^ sources promote damage and others signaling, and that the outcome changes depending upon mitochondrial energetic state and the levels and functional state of O_2_^.−^ and H_2_O_2_ reactants. Concordantly, Bleier et al. (2015) documented a differential pattern of target protein thiol modification when ETC I O_2_^.−^ production was induced compared to ETC III. That is, the identity of the protein thiol modified depends on the site of O_2_^.−^ generation. Analogously, O_2_^.−^ and subsequent H_2_O_2_ generated by one source may damage mitochondrial DNA whereas another may not. Elucidating the site (s) that damage mitochondrial DNA would significantly advance current understanding. Fate 5 is intriguing because H_2_O_2_ diffusing out of the mitochondrion could function as a retrograde nuclear signal (Balaban et al., 2005; Murphy, 2009, 2012). This could involve direct or indirect (redox relay) H_2_O_2_ induced modification of redox-sensitive transcription factors and resultant regulation of nuclear transcription (Murphy, 2009). It could equally involve nuclear ^·^OH generation and consequent DNA damage, the sensing of which could also alter cell signaling processes (see main text).

## Exercise-induced redox signaling: The key role of hydrogen peroxide

Exercise-induced ^·^OH mediated DNA damage proceeds in a random and indiscriminate chemical manner, exemplified by a myriad of guanine oxidation products (Radak et al., 2011b). Exercise-induced redox signaling, however, depends on the transduction of specific, reversible and compartmentalized chemical signals (Cobley et al., 2015a). ^·^OH is chemically unable to transmit a signal in this conventional manner (Holmstrom and Finkel, 2014). Hence, exercise-induced DNA damage and redox signaling are not necessarily mediated by the same species. Mechanistic knowledge of exercise-induced redox signaling is fragmentary (Cobley et al., 2015a). For instance, reversible cysteine oxidation is a key feature of redox signaling (Go and Jones, 2013). The proteome contains 214,000 cysteine residues, with ~21,000–40,000 modified following addition of oxidizing stimuli (Jones, 2008). If one assumes that a lower limit of ~21,000 cysteine residues are exercise-responsive then ≤1% of the exercise-responsive cysteine proteome has been mapped to date. Indeed, we are unaware of any cysteine based exercise proteomics study. Insights from the parent discipline (i.e., redox biology) are therefore, utilized to provide a brief chemical synopsis of the likely nature of exercise-induced redox signaling.

Current redox signaling paradigms are defined by specific and reversible target protein cysteine modifications that alter protein activity, partner binding and location (Janssen-Heininger et al., 2008; Winterbourn, 2008; Forman et al., 2014a,b). Notable modifications include disulfide, mixed disulfide formation and S-Nitrosylation (Gallogy and Mieyal, 2007; Benhar et al., 2009; Poole, 2015). Redox signaling is thought to be predominately mediated by two electron oxidants, with H_2_O_2_ considered to be one of the few species capable of selective reaction with reactive cysteine residues on target proteins (Forman et al., 2010). The direct reaction of H_2_O_2_ with often low abundant signaling proteins (e.g., KEAP1 estimated: *k* ~ 140 M^−1^ s^−1^; Marhino et al., 2014) must compete against the rapid reaction of H_2_O_2_ with several highly abundant redox enzymes (e.g., peroxiredoxins [Prx]: *k* ~ 10^5^–10^8^ M^−1^ s^−1^; Brigelius-Flohe and Flohe, 2011; Karplus, 2015). Further, acute exercise increases the activity of redox enzymes, notably catalase (Powers and Jackson, 2008). How H_2_O_2_ overcomes this kinetic bottleneck is a matter of debate, but could involve redox relays, wherein oxidizing equivalents are transferred from the antioxidant enzyme to the signaling protein (Marhino et al., 2014). For instance, the reaction of H_2_O_2_ with Prx II has recently been shown to be coupled to the oxidation of STAT3 (Sobatto et al., 2015). High local H_2_O_2_ gradients in specific cellular compartments (co-localization of target and source) allied to sequestration and/or inactivation of antioxidant enzymes may also facilitate direct H_2_O_2_ signaling (Woo et al., 2010; Marhino et al., 2014). Analogous to exercise, many unanswered questions remain regarding the precise chemical nature and spatiotemporal regulation of redox signaling (Brigelius-Flohe and Flohe, 2011; Levonen et al., 2014). It follows that one cannot fully appraise the interplay between exercise-induced redox signaling and DNA damage, since many mechanistic details have yet to be elucidated.

## Interplay between exercise-induced DNA damage and redox signaling: A nuanced view

Chemical delineation of the species and reactions responsible for DNA damage and redox signaling is necessary from a mechanistic perspective (Buettner, 2015; Forman et al., 2015) but one should not view the two processes as wholly discrete and independent. Although redox signaling can occur without oxidative damage and vice versa (Jones, 2008; Jones and Go, 2010) the two processes can be interlinked in several hitherto underappreciated ways, in an exercise setting. Possible points of interaction will be appraised herein.

## H_2_O_2_: An upstream bifurcation point

We hypothesize that H_2_O_2_ acts as a bifurcation point between exercise-induced redox signaling and damage. A key feature of our hypothesis is that the H_2_O_2_ “interactome” does not trap all of the H_2_O_2_ generated at signaling sites during exercise, permitting distal diffusion and the induction of DNA damage following reaction with transition metals (Reactions 1, 2). In this model, ^·^OH mediated DNA damage is a by-product of exercise-induced redox signaling. To illustrate this model an exemplar scenario is next considered (see Box 1 for a mitochondrial scenario).

**Figure 1:**
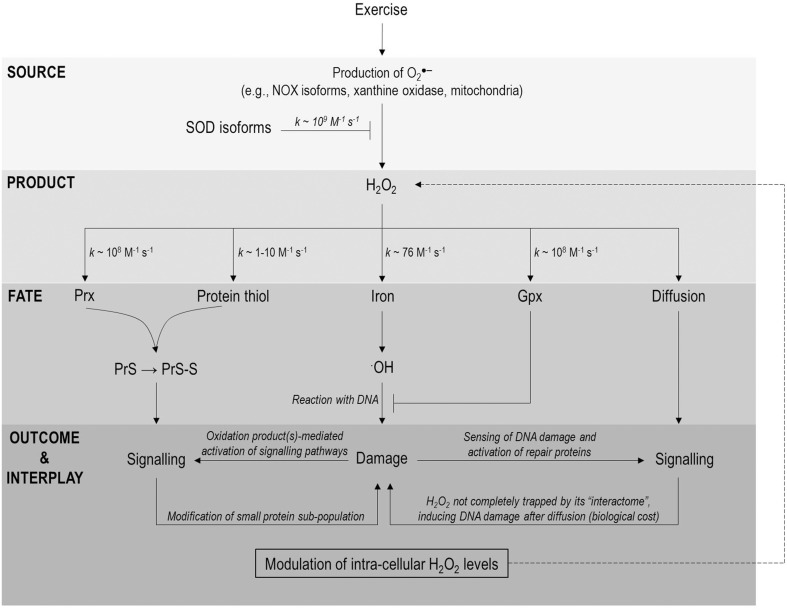
**A H_2_O_2_ biological fate flow chart highlighting possible points of interaction between exercise-induced DNA damage and redox signaling**. Exercise increases O_2_^.−^ generation from a variety of sources (e.g., NOX isoforms, xanthine oxidase and mitochondria) which can be converted to H_2_O_2_ in a reaction catalyzed by SOD isoforms. Once formed H_2_O_2_ has 5 principal fates (1) reaction with Prx isoforms and oxidation of a signaling protein via a redox relay (2) direct reaction with a signaling protein (3) reaction with iron (other metals not shown for clarity) and ^·^OH generation (4) reaction with a protective redox enzyme (e.g., Gpx1) and (5) diffusion from source (permitting any of fates 1–4). Note the protective function of Prx and indeed catalase are not shown for the purposes of clarity. Fates 1–2 can lead to target protein modification and thus redox signaling whereas fate 3 can underpin DNA damage following the indiscriminate, diffusion-controlled ^·^OH-DNA reaction. Signaling and damage may be linked in two important ways from there (1) modification of redox repair enzymes and (2) modulation of H_2_O_2_ levels by redox repair enzymes (depicted a dashed line). Note many of these links have yet to be documented in an exercise setting and are thus speculative at present.

NADPH oxidase (NOX) isoforms appear to be the principal intracellular source of O_2_^.−^ during exercise (Sakellariou et al., 2013). Prototypical models of redox signaling are defined by activation of plasma membrane bound NOX, secondary to Rac1 recruitment, and growth factor stimulation (Brandes et al., 2014; Holmstrom and Finkel, 2014). Note NOXs are likely activated by different stimuli (e.g., Ca^2+^ fluxes) during exercise (Sakellariou et al., 2014) and are redox regulated (Brandes et al., 2014). NOX generate extracellular O_2_^.−^ (Reaction 3) which can spontaneously dismutate to H_2_O_2_ (*k* ~ 10^5^ M^−1^ s^−1^; Forman et al., 2010) or be converted to H_2_O_2_ enzymatically in a reaction catalyzed by superoxide dismutase (SOD) isoforms (*k* ~ 10^9^ M^−1^ s^−1^; Reaction 4, McCord and Fridovich, 1969). H_2_O_2_ can re-enter the cell via diffusion or though specialized aquaporin/peroxiporin channels (Bienert and Chamont, 2014; Sies, 2014). Thereafter, the topology of the plasma membrane permits spatial gating with lipid rafts and caveolae providing a means of regulating and channeling signals in discrete membrane domains (Patel and Insel, 2009). The relatively slow reaction of H_2_O_2_ with even highly reactive cysteine residues on target signaling proteins (*k* ~ 1–10 M^−1^ s^−1^; Winterbourn and Hampton, 2008) could therefore, be compensated for by “insulating” the signal against other reactants and increasing local concentrations of target and reactant (Forman et al., 2014b). Intricate spatial regulation is demonstrated by the observation that redox signaling proceeds with modification of only a small protein sub-population (Sobatto et al., 2015). Evidenced in the H_2_O_2_ → Prx II → STAT3 redox relay wherein only a fraction of total STAT3 is modified and thus exhibits reduced transcriptional activity (Sobatto et al., 2015). This microenvironment may facilitate proximal trapping of signal and target, perhaps by lipid barriers restricting say the lateral diffusion of H_2_O_2_. Nevertheless, some H_2_O_2_ diffusion is likely and this might have unwanted distal effects (e.g., DNA oxidation, see Figure 1). DNA oxidation may, therefore, be one of the biological “costs” of H_2_O_2_ signaling and may explain why H_2_O_2_ is toxic to cells, at even micromolar concentrations (Nakamura et al., 2003). Alternatively, DNA oxidation secondary to signaling may not be “costly” at all, but rather an indirect redox-sensing mechanism, providing information on cellular redox state via sensing of a terminal product (discussed below).

*Reaction 3, NOX reaction*: 2O_2_ + NADPH → NADP^+^ + H^+^ + 2O_2_^.−^
*Reaction 4, SOD reaction*: 2O_2_^.−^ + 2H^+^ → H_2_O_2_ + O_2_

The explanatory power of the bifurcation hypothesis, whilst cogent is conceptual in an exercise setting at present. It is, therefore, necessary to consider evidence for any distal reactions that could damage DNA. Measuring and deciphering the origin of nuclear ^·^OH is complicated by several technical issues (Halliwell and Whiteman, 2004). Although, direct evidence is lacking, redox signaling is commonly associated with the oxidation of the intracellular DCFH probe to DCF (Winterbourn, 2015). We emphasize that the DCFH assay does not measure H_2_O_2_ (no direct H_2_O_2_-DCFH reaction) and is oxidized by one electron mechanisms (Wardman, 2007; Winterbourn, 2014). Further the assay is prone to several artifacts, notably spurious amplification of the signal, via rapid reaction of the DCF radical intermediate with O_2_ (*k* ~ 10^8^ M^−1^ s^−1^) to yield O_2_^.−^ (Kalyanaraman et al., 2012; Winterbourn, 2014). Despite several caveats Kalyanaraman et al. (2012) note that the DCFH assay can provide valuable information on redox dependent iron signaling (Tampo et al., 2003). The temporal association of redox signaling and DCFH oxidation could, in part, reflect the efflux of H_2_O_2_ from signaling microdomains, subsequent reaction with iron and thus DNA oxidation. It could equally reflect an underappreciated role of free radicals in redox signaling, likely via the generation of thiyl radicals (Winterbourn and Hampton, 2008; Winterbourn, 2015). In any event, we postulate that exercise-induced redox perturbations are bi-functional, resulting in signaling and damage, and that the fate of H_2_O_2_ can, in part, regulate the extent of each outcome.

## Sensing DNA damage: Implications for redox signaling

Conceptually, DNA oxidation may influence redox signaling directly through DNA oxidation product (s) and/or indirectly through the redox regulation of proteins implicated in DNA repair (Radak et al., 2013). Each possibility will next be considered in turn with exemplars provided.

Oxidized macromolecule adducts are not chemically inert (Niki, 2009) and are thus not a passive end-point of exercise-induced reactive species generation. Indeed, many oxidized macromolecule adducts are biologically active and can influence cell signaling processes (Brigelius-Flohe and Flohe, 2011). A proof-of-concept example is the participation of lipid peroxidation products in Nrf-2-KEAP-1 signaling (Niki, 2012). The Nrf-2-KEAP-1 pathway regulates the expression of cyto-protective genes (e.g., hem oxygenase; Kasper et al., 2009). In the inactive state, KEAP-1 sequesters Nrf-2 in the cytoplasm, facilitating the E3 ubiquitin ligase cullin-3 mediated Nrf-2 proteolysis (Levonen et al., 2014). This inhibition can be relieved by S-alkylation and the subsequent degradation of KEAP-1, enabling the nuclear translocation of Nrf-2 (Forman et al., 2014a). KEAP-1 S-alkylation can be mediated by lipid peroxidation products, such as 4-hydroxy-2-noneneal (Chen et al., 2005, 2006). In considering DNA oxidation products, application of exogenous 8-oxo-G to cells alters signaling (Aguilera-Aguirre et al., 2015). This is, however, likely mediated by the binding of 8-oxo-G to DNA repair proteins and not post-translational modification (Aguilera-Aguirre et al., 2015). Indeed, to the best of our knowledge there are no examples of DNA oxidation products directly altering the post-translational modification state of signaling proteins. This notwithstanding, free 8-oxo-G can be oxidized to a hydroperoxide like derivative that could signal, but this remains speculative at present (Hajas et al., 2012). Altogether, the possibility that DNA damage products directly participate in signaling reactions is not excluded but is not presently an example of the interplay between exercise-induced oxidative damage and signaling and is thus not considered further.

The consequences of oxidative damage, genomic rearrangements, and strand breaks, are sensed by repair proteins (Dizdaroglu, 2012). For example, 8-oxoG is excised by OGG1 a key component of the mitochondrial and nuclear base excision repair pathway (Radak et al., 2011b). OGG1 is regulated by a plethora of post-translational modifications, including redox-regulated disulfide formation, which is associated with reduced OGG1 activity (Bravard et al., 2006). Indeed, several other proteins implicated in DNA repair including but not limited to SIRT1 (Hwang et al., 2013), SIRT6 (Hu et al., 2015), and p53 (Malliet and Pervaiz, 2012) are also redox regulated. Redox signaling has the capacity therefore, to influence DNA repair and could constitute one convergence point between exercise-induced DNA damage and redox signaling. Another point is provided by the interaction of OGG1 with Rac1 (Hajas et al., 2013). Rac1 is a small GTPase that is regulated by GTP loading, being active in the GTP but not the GDP bound state (Bosco et al., 2009). Rac1 GTP loading is redox-sensitive being regulated by cys18 oxidation and S-glutathionylation (Heo and Campbell, 2005; Hobbs et al., 2015). Rac1, in turn, regulates cellular redox state in several ways, notably through binding and activating NOX isoforms (Leto et al., 2009; Nauseef, 2014). Interestingly, 8-oxoG bound OGG1 complexes can bind Rac1 and promote GTP loading and subsequent NOX4 activation following a rise in 8-oxo-G levels (Hajas et al., 2013). This is associated with increased intracellular H_2_O_2_ probe and general redox indicator probe DCFH oxidation (Hajas et al., 2013). This interaction provides a mechanistic link between redox signaling and damage that may constitute a feedback loop. The functionality of this feedback loop is, however, unclear and remains to be investigated in an exercise setting. Overall, two points of interplay are apparent: (1) the redox regulation of DNA repair proteins and (2) the capacity of DNA repair proteins to modulate intracellular H_2_O_2_ levels and perhaps redox signaling and damage.

## Concluding perspectives

The terminal reactions that define exercise-induced ^·^OH mediated DNA damage and H_2_O_2_ mediated signaling are chemically distinct yet we have delineated possible points of interaction between the two processes. Of course, redox signaling can proceed without DNA damage and vice versa (Jones, 2006, 2008). Indeed, this paradigm is well-established in many settings and likely occurs with the nanomolar (~10–100 nM; Levonen et al., 2014) H_2_O_2_ fluxes that define growth factor signaling in the resting state (Rhee, 2006). Exercise-induced quantal H_2_O_2_ yields are likely in the micromolar range (~1 μM; Palomero et al., 2008) and in this situation DNA damage and redox signaling are unlikely to be mutually exclusive. We postulate that the biological fate of H_2_O_2_ represents a bifurcation point that, in part, delineates the extent of exercise-induced DNA damage and signaling. In this scenario, crosstalk between redox signaling and DNA damage is facilitated by (1) the redox regulation of DNA repair proteins (e.g., OGG1; Bravard et al., 2006) and (2) the capacity of DNA repair proteins to modulate intracellular H_2_O_2_ levels (OGG1-Rac1-Nox4) axis (Hajas et al., 2013). Perhaps exercise-induced H_2_O_2_ levels define an interface between redox responses that are typically, but not always specific “on/off switches” (e.g., kinase activation) and general rheostats (e.g., repair processes that “sense” DNA damage). This hypothesis may have considerable explanatory power. The dual functionality of exercise-induced H_2_O_2_ fluxes is consistent with the temporal co-incidence of the redox-regulation of signaling proteins (e.g., PGC-1α, Kang et al., 2009) and generalized signaling responses to macromolecule damage (e.g., apoptosis secondary to DNA oxidation, Winterbourn, personal communication). Saliently, a generalized signaling response need not require reactive species to signal in a classical way, akin to a phosphorylation cascade. Rather, it simply requires the sensing of a redox perturbation at a critical juncture: DNA oxidation. This discourse may provide a mechanistic framework to further explain how acute exercise-induced DNA damage acts as an adaptive signal to stimulate protection against exercise-induced genomic stress. It is emphasized that other points of interaction may exist but were not considered owing to space constraints. Indeed, the biological fate of peroxynitrite may constitute another salient bifurcation point. Ultimately, this dialog is intended to stimulate further investigation into the mechanisms regulating exercise-induced redox signaling and damage.

## Author contributions

JC conceived, drafted and edited the manuscript. NM and MN conceived the figures and drafted sub-sections of the manuscript. All authors (JC, NM, JPM, GC, MN, and JKM) critically edited and approved the final manuscript.

### Conflict of interest statement

The authors declare that the research was conducted in the absence of any commercial or financial relationships that could be construed as a potential conflict of interest.
